# Prevalence and Correlates of Hepatitis B and C infections in Sickle cell anaemic (SCA) children compared to Controls in a Tertiary Hospital, Abakaliki, Southeast, Nigeria

**DOI:** 10.4314/ahs.v25i1.13

**Published:** 2025-03

**Authors:** Samuel Amechi Nwukor, Chinonyelum Thecla Ezeonu, Maria-lauretta Chito Orji, Patricia Ngozi Udechukwu, Nnaemeka Kenneth Omeje

**Affiliations:** Department of Paediatrics, Federal University Teaching Hospital, Abakaliki, Ebonyi, State

**Keywords:** Case-controls, Correlates, Hepatitis B virus, Hepatitis C virus, Prevalence, Sickle cell anaemia

## Abstract

**Background:**

Practices among sickle cell anaemic (SCA) children may increase their risk of infection with blood-borne viruses. The study aimed to determine the prevalence and correlates of hepatitis B virus (HBV) and hepatitis C virus (HCV) infections among children with SCA compared to controls.

**Methods:**

A hospital-based cross-sectional study that involved 200 children with SCA and 200 non-SCA children (controls). Information was obtained using an interviewer-administered questionnaire. Screening for hepatitis B surface antigen (HBsAg) and anti-hepatitis C virus (anti-HCV) was done using Smartcare® rapid diagnostic tests (RDT) kits for HBV and HCV. Data was analyzed using the Statistical Package for Social Science (SPSS) version 22. P<0.05 was considered statistically significant.

**Results:**

Five (2.5%) and 21 (10.5%) of the SCA children recruited into the study were positive for HBsAg and anti-HCV respectively, compared to 2(1.0%) and 13 (6.5%) observed in controls. The Logistic regression analysis revealed that female gender (AOR=3.39, 95%CI=1.20-9.57, p=0.016), age (AOR=0.88, 95%CI=0.79-0.99, p=0.033), and multiple ear piercings (AOR=1.93, 95%CI=1.17-21.59, p=0.021) were correlates of HCV infection among study participants.

**Conclusion:**

A high prevalence rate of HCV infection was observed among children with SCA and was significantly associated with a modifiable variable.

## Introduction

Hepatitis B and C infections occur globally and are seen in all age groups. Prevalence rates in countries in Africa range from 5% -15%^1^. A systematic review and meta-analysis by Musa et al^2^ in Nigeria reported the pooled prevalence of HBV at 13.6% with a range of 0.5 – 46.8%. Hepatitis C virus infection is still under-diagnosed and underreported in Africa aside from Egypt with a prevalence of 17.5%^3^. The prevalence of HCV in the general population in the region of Africa is about 0.1% -17.5%. In Nigeria, studies done in Ilorin^4^, Enugu^5^, and Benin^6^ showed an increasing prevalence of HCV from 3.0%, 6.6%, and 20% respectively.

Blood-borne viral infections such as HBV, HCV, and HIV are readily transmitted from infected blood during transfusion, sexual contact with an infected person, use of unsterilized sharp objects for scarification marks, tattooing, and ear piercing with contaminated instruments^7^. Children with sickle cell anaemia, are prone to frequent and recurrent multiple crises, recurrent hospital admissions with attendant injections, and frequent blood transfusions as well^8^. Occasionally, treatments are received from unskilled healthcare providers who give medications with contaminated needles^9^. Frequent treatments involving blood transfusions even from standard centres, have the potential to bring about HBV transmissions to susceptible recipients.^6^ However, previous studies^10^,^11^ observed no significant relationship between blood transfusion and the prevalence of HBV and HCV infections among study participants. They opined that the robust safety protocol ensured in the blood transfusion process in health centers may have influenced their finding. The cultural practice of ‘bloodletting of bad blood’ on sickle cell anaemic children in the bid to cure the disease, often leaves scarification marks on the chest wall where it is performed. This practice is usually done with unsterilized instruments^12^. Other socio-cultural practices such as tattooing, fixing artificial eyelashes, and multiple ear piercings with contaminated objects increase the risk of infection with blood-borne viruses^10^.

The prevalence of Hepatitis B infections among sickle cell anaemia children in Nigeria varies significantly, ranging from 2.4% to 17.5%, depending on the sample size used and variables considered among the study participants. While some of the studies^13^,^14^ noted frequency of blood transfusion as a determinant of the acquisition of HBV among SCA children, others did not^15^.

A case-control study by Bolarinwa et al^16^ in Ile-Ife, South West, Nigeria reported a seroprevalence of HBV infection in SCA as 2.4% in comparison with seroprevalence of 2.2% among controls. The study compared transfused and non-transfused subjects and controls. The result was not statistically significant, since blood transfusion did not significantly influence the risk of HBV infection. This study reaffirms the study by Okocha et al^17^ that blood transfusion did not increase significantly the infection rate of HBV infection. However, the limitation of the study by Bolarinwa et al^16^ is the small sample size of 82 confirmed children with SCA, hence a low study power.

A study by Onuchukwu et al^4^ in Ilorin Teaching Hospital (UITH) Ilorin involving 82 transfused SCA and 84 non-transfused SCA children; reported a 3.7% seroprevalence rate of anti-HCV in the transfused sickle cell anaemia (SCA) children and 2.4% in the controls group using second generation ELISA methods. More males were affected than females probably because they are prone to more crises and hence more transfusion because of more adventures. This again had a small sample size. A cross-sectional survey of 512 subjects from rural China by Yin et al 18 revealed that HCV RNA positivity was found in 13.8% of the participants, factors were blood transfusions, toothbrush and razor blade sharing, acupuncture, therapeutic injection, and blood donations for monetary gain represented the highest risk factor. There is a dearth of information on possible determinants of Hepatitis B and C infections among SCA children in Ebonyi state and the Southeastern part of Nigeria. Therefore, this study aims to determine the prevalence and predictors of hepatitis B and C viral infections among SCA children in Southeastern, Nigeria. Hopefully, the evidence found in this study will be beneficial in health education, and awareness creation of the risk factors of HBV and HCV infections among children, especially those with SCA.

## Materials and Methods

It was a hospital-based, cross-sectional comparative study carried out between May and September 2020. The study was conducted in the Sickle Cell Centre and Children Out Patient Clinic of Alex Ekwueme Federal University Teaching Hospital, Abakaliki (AE-FUTHA) in Ebonyi State, South-east Nigeria. This hospital is situated in Abakaliki metropolis, the capital city of Ebonyi State, Southeast Nigeria.

### Sample Size

The sample size was calculated using Pocock's formula^19^ for determining sample size involving two groups in a population. The reported prevalence rates of 17.3% for HBsAg, and 12.7 for anti-HCV^20^ were used in calculating the sample size. Assuming a power of 80%, a confidence interval of 95%, a minimum sample size of approximately 200, and a control group of 200, age and gender-matched non-SCA children (Hb AA and Hb AS) were also recruited into the study.

### Subject selection

All children aged 6 months to 17 years with confirmed SCA (Hb SS) attending the Paediatric Sickle Cell Clinic of Alex Ekwueme Federal University Teaching Hospital, Abakaliki, whose caregivers gave informed written consent were consecutively recruited into the study until the sample size was met. Haemoglobin (Hb) electrophoresis was carried out, for children of consenting parents who came to the Children Out-patient Clinic of Alex Ekwueme Federal University Teaching Hospital, Abakaliki for minor ailments or routine medical fitness examination, children who were found to be hemoglobin AA (Hb AA) or Haemoglobin AS (Hb AS) and had no evidence of critical illness such as high temperature, respiratory distress, severe pallor, and severe jaundice, was included into the study as controls. The socioeconomic status of the participants was determined using the method described by Olusanya et al^21^, which classifies socioeconomic class into upper, middle, and lower classes using the father's occupation and the mother's literacy level.

### Sample collection

Each participant was reassured of minimal pain, a separate room was dedicated for blood sample collection to avoid the generation of fear and anxiety in the other participants and controls during blood sampling. The tourniquet was tied above the elbow to make the antecubital veins visible and prominent; the antecubital fossa was properly cleaned with the swab soaked in the methylated spirit. Five milliliters of venous blood was drawn from each of the participants with SCA and controls and sent to the laboratory in an EDTA container for sample analysis. The samples were mixed well to prevent clotting and labeled with unique identification numbers. The blood was centrifuged within 90 minutes of collection and the plasma was kept in a refrigerator below -20 degrees Celsius pending when all the samples for the day were collected. Daily sample collection was at a range of 5 to 10 and screening for HBsAg and anti-HCV using Smartcare rapid diagnostic tests (RDT) kits for HBV and HCV respectively were carried out within 6 hours of sample collection. The RDT kits used for HBsAg and anti-HCV assay were produced by ACON Laboratories Inc, USA, San Diego, S, n, R015039 (Smartcare®). The kits were stored in the temperature range of 4–30 degrees Celsius in the laboratory refrigerator.

### Quality control

The Smartcare® test kit was checked for expiration and for proper sealing. A procedural control technique was included in the assay and the presence of a red line appearing in the control point (C) is the internal procedural control. The test was reported as positive, negative, and invalid. For invalid test results, another sample was collected and the test was repeated. All children who tested positive for HBV and HCV were properly counseled on the nature of the disease and referred to the gastroenterology clinic of the hospital for expert care.

### Operational definitions

Cases: Sickle cell anaemic children with Hb SS genotype.

**Controls:** Children with Hb AA and Hb AS genotype

Blood-borne viral infections are infections transmitted through blood and blood products, these include HIV, hepatitis B, C, and E, and syphilis. This study delved into HBV and HCV infections.

## Ethical Considerations

Ethical approval was obtained from Alex Ekwueme Federal University Teaching Hospital Abakaliki Research Ethics Committee. Informed written consent was sought from the guardians of all the participants and assent from the participants from 7 years and above.

### Data analysis

Data was collected utilizing a structured questionnaire for each study participant. The data was analyzed using the software package for Social Science (SPSS) 22.0 for Windows. Data presentation was done by frequency tables and charts. The relationship between categorical variables was analyzed using the Chi-square test and the Fisher exact test. The latter was used in situations in which the Chi-square test could not be used, such as with small values in cells less than 5. A logistic regression model was used to test the level of association between HBV and HCV, and predictors of infections. The level of statistical significance was kept at p≤ 0.05.

## Results

An equal number of cases and controls aged 6 months to 17 years were recruited into the study; each comprising 200 participants. Among the cases, 114 (57%) were males with a male-to-female ratio of 1.3:1.0. The median (IQR) age for both groups was 10.0 (10.0) years. The majority of the SCA (71%) and controls (72%) dwelt in urban settlements. [Table 1]

**Table 1:** Socio-demographic profile of children with SCA and controls

Variables	Cases N = 200 (%)	Controls N = 200 (%)
**Median Age (IQR)**	10.00 (10.00)	10.00 (10.00)
**Age group**		
6months-1year	3 (1.5)	3 (1.5)
2-5 years	49 (24.5)	49 (24.5)
6-12 years	69 (34.5)	69 (34.5)
13-17 years	79 (39.5)	79 (39.5)
**Gender**		
Male	114 (57.0)	113 (56.5)
Female	86 (43.0)	87 (43.5)
**Area of residence**		
Urban	142 (71.0)	144 (72.0)
Rural	58 (29.0)	56 (28.0)
**Social class**		
Upper	80 (40.0)	111 (55.5)
Middle	51 (25.5)	49 (24.5)
Lower	69 (34.5)	40 (20.0)

The prevalence of hepatitis B virus infection among SCA was noted to be 2.5% (5/200) compared to the controls, with a prevalence rate of 1.0% (2/200). The difference was not significant. A total of 21 (10.5%) of the 200 children withSCA had HCV infection while 13 (6.5%)c hildren from th e control group were positive for the same infection. The difference in prevalence rate was also not significant as shown in Table 2.

**Table 2:** Comparison of the prevalence of HBV and HCV infections between cases and controls

Variables	Cases n (%)	Controls n (%)	*χ* ^2^	p-value
**Hepatitis d virus infection**				
Positive	5 (2.5)	2 (1.0)		
Negative	195 (97.5)	198 (99.0)	1.56	0.450
**Total**	200 (100.0)	200 (100.0)		
**Hepatitis C virus infection**				
Positive	21 (10.5)	13(6.5)		
Negative	179 (89.5)	187 (93.5)	0.46	1.790
**Total**	200 (100.0)	200 (100.0)		

**Figure 1:**
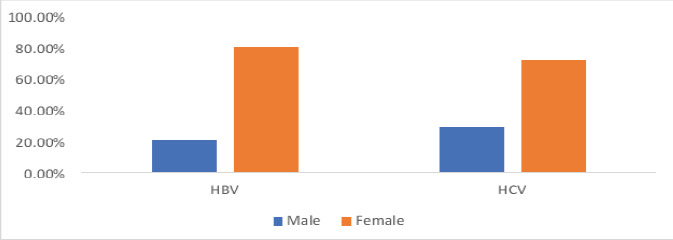
Gender representation of the prevalence rates of HBV and HCV infections in SCA children

Figure 1 shows that more females than males were infected with HBV and HCV infections. A total of 4 (80.0%) of the 5 SCA children with HBV infection and 15 (71.4%) of the HCV infection in SCA children were females.

The median age for SCA children with hepatitis B virus infection was 14 years (IQR= 3 years) compared to 10 years (IQR=10 years) seen in SCA without HBV infection. This age difference was noted to be statistically significant (<0.001). [Table 3] More of the SCA children dwelling in rural settlements (80%, 4/5) had the HBV infection compared to 20% (1/5) found in urban dwellers. Of note was that none of the SCA children who received blood transfusion in a hospital setting (75/118, 63.6%) had HBV infection. There were statistically significant relationships between place of dwelling and acquisition of HBV infection (p=0.030). [Table 3]

**Table 3:** Bivariate analysis between socio-demographic, clinical, and lifestyle factors with HBV infection among children with sickle cell anaemia

Variables	Hepatitis B Virus Infection		Test Stat	p-value	Crude Odd Ratio
	
	Positive n (%)	Negative n (%)			(95% CI)
**Median Age in Years**	14.00(3.00)	10.00(10.00)	U =131.0	<0.001	4.17 (2.01-6.32)
**(IQR)**					
**Gender**			FT	0.170	-
Male	1 (20.0)	113 (57.9)			
Female	4 (80.0)	82			
		(42.1)			
**Area of Residence**					
Urban	1 (20.0)	141 (72.3)			
Rural	4 (80.0)	54 (27.7)	FT	0.030	10.44 (1.14-95.56)
**Social Class^21^**			*χ*^2^ = 0.98	0.61	-
Upper	1 (20.0)	79 (40.5)			
Middle	2 (40.0)	49 (25.1)			
Lower	2 (40.0)	67 (34.4)			
**HBV Vaccination**					-
Yes	3 (60.0)	180 (92.3)	FT	0.060	
No	2 (40.0)	15 (7.7)			
**History of Blood**					-
**Transfusion**					
Yes	2 (40.0)	118 (60.5)	FT	0.390	
No	3 (60.0)	77 (39.5)			
**Place of blood transfusion**					
Tertiary hospitals	0 (0.0)	75 (63.6)	FT	0.189	
Non-tertiary hospitals	2 (100.0)	43 (36.4)			
**History of parenteral injection**	5 (100.0)	167 (85.6)	FT	0.660	-
Yes	0 (0.0)	28 (14.4)			
No					
**Tattoo/scarification marks**	1 (20.0)	17 (8.7)	FT	0.380	-
Yes	4 (80.0)	178 (91.3)			
No					
**Sexually active**			FT	1.000	-
Yes	0 (0.0)	6(3.1)			
No	5 (100.0)	189 (96.9)			
**Multiple ear piercings**	4 (80.0)	82 (42.1)	FT	0.170	-
Yes	1 (20.0)	113 (57.9)			
No					
**Local uvulectomy**			FT	1.000	-
Yes	0 (0.0)	3 (1.5)			
No	5 (100.0)	192 (98.5)			

U = Mann-Whitney U-test; FT = Fisher exact Test, IQR = Interquartile Range, CI = Confidence Interval

The median age for HCV infection among SCA children was 14 years, with more females (71.4%, 15/21) having the infection. Being a female (*χ*^2^ = 7.74, p=0.005), having tattoos or scarification marks (*χ*^2^ = 6.28, p=0.010), having a history of uvulectomy (FT, p=0.030), and engaging in multiple ear piercings (*χ*^2^ = 7.74, p=0.005) had significant relationships with the acquisition of HCV infection [Table 4]

Table 5 shows the Logistic regression analysis to ascertain the independent risk for HCV infection. Female gender (AOR= 3.39, 95%CI= 1.20-9.57, p=0.016), age (AOR= 0.88, 95%CI= 0.79-0.99, p=0.033), and multiple ear piercings (AOR= 1.93, 95%CI= 1.17-21.59, p=0.021) were found to be independent risk factors to HCV infections in SCA children. [Table 5]

**Table 4:** Bivariate analysis between socio-demographic, clinical, and lifestyle factors with HCV infection among children with sickle cell anaemia

Variables	Hepatitis C Virus Infection		Test Stat	p-value	Crude Odd Ratio
	
	Positive n (%)	Negative n (%)			(95% CI)
**Median Age in Years**	14.00(5.00)	9.00(10.00)	U =141.00	0.004	3.07 (2.01-5.32)
**(IQR)**					
**Gender**					
Male	6 (28.6)	108 (60.3)			
Female	15 (71.4)	71 (39.7)	*χ*^2^ = 7.74	0.005	3.80 (1.41 – 10.27)
**Area of Residence**					-
Urban	13 (61.9)	129 (72.1)	*χ*^2^ = 0.94	0.330	
Rural	8 (38.1)	50 (27.9)			
**Social Class**					
Upper	6 (28.6)	74 (41.3)	*χ*^2^ = 1.96	0.380	-
Middle	5 (23.8)	46 (25.7)			
Lower	10 (47.6)	59 (33.0)			
**History of Blood Transfusion**	12 (57.1)	108 (60.3)	*χ*^2^ = 0.08	0.790	-
Yes No	9 (42.9)	71 (39.7)			
**Place of blood transfusion**					
Tertiary hospitals	5 (41.7)	70 (64.8)			
Non-tertiary hospitals	7 (58.3)	38 (35.2)	*χ*^2^ = 0.12	0.462	
**History of parenteral injection**	20 (95.2)	152 (84.9)			
Yes No	1 (4.8)	27 (15.1)	FT	0.430	-
**Tattoo/scarification marks**	5 (23.8)	13 (7.3)	*χ*^2^ = 6.28	0.010	3.99 (1.26–12.63)
Yes No	16 (76.2)	166 (92.7)			
**Sexually active**					
Yes No	1 (4.8) 20 (95.2)	5 (2.8) 174 (97.2)	FT	0.490	-
**Multiple ear piercings**					
Yes No	15 (71.4) 6 (28.6)	71 (39.7) 108 (60.3)	*χ*^2^ = 7.74	0.005	3.80 (1.41 – 10.27)
**Local uvulectomy**					
Yes No	2 (9.5) 19 (90.5)	1 (0.6) 178 (99.4)	FT	0.030	18.74 (1.62 – 216.41)

U = Mann-Whitney U-test; FT = Fisher exact test, IQR = Interquartile Range, CI = Confidence Interval.

**Table 5:** Logistic regression to determine the independent predictors of HCV infection among the cases

Independent Predictors	Adjusted Odd Ratio (AOR)	95%CI	p-value
**Age**	0.88	0.79-0.99	0.033
**Gender**			
Female	3.39	1.20 – 9.57	0.016
Male			
**Tattoo/scarification**			
Yes	0.27	0.07 – 1.04	0.056
No			
**Multiple ear piercings**			
Yes	1.93	1.17 – 21.59	0.021
No			
**Local uvulectomy**			
Yes	0.23	0.02-3.18	0.270
No			

Dependent variable = Hepatitis C infection categorized as 0= non-reactive and 1= reactive

## Discussion

The study showed HBsAg prevalence of 2.5% for children with SCA and 1% for the controls. The prevalence rate of anti-HCV was 10.5% and 6.5% for the children with SCA and the controls respectively. While the risk of HBV infection was observed to be higher among adolescents and rural dwellers, HCV infection was higher in the adolescent age bracket, female gender, presence of scarification marks/tattoo, multiple ear piercings, and local uvulectomy. However, age, female gender, and multiple ear piercings were significantly associated with HCV infection. The female gender and engaging in multiple ear piercings increased the risk of acquiring HCV infection thrice and twice respectively among children with SCA. The low prevalence of HBsAg in this study group could be ascribed to the fact that the majority of the cases that received blood transfusion had them in the tertiary hospital where there are readily available blood banking services and strict compliance with screening for transfusion-related infections as seen in some studies^13^,^15^. Furthermore, the high immunization coverage (92.3%) with the HBV vaccine observed in this study, may have also contributed to the low prevalence of HBsAg in cases and controls. This is in agreement with the findings by Uleanya and Obidike^22^, Babatola et al^11^ and Ikobah et al^23^ who pointed out the effectiveness of HBV vaccination in their studies. This study showed low levels of risky practices like sexual activity, non-abuse of any illicit drugs among the cases, and a low rate of female circumcision, which may have influenced the low prevalence of HBV infection. The low prevalence of HBsAg in this study was similar to studies by Bolarinwa et al^16^ 2.4% in Ile Ife, Aliyu et al^24^ 2.0% in Zaria, George and Yaguo Ide^13^ 3.6% in Port Harcourt, Mehri et al^25^ 1.8% in Saudi Arabia, Al-Kadassy et al^26^ 3.3% in Yemen. On the contrary, Sadoh and Ofili^27^ reported a high prevalence rate of 13.9% despite high immunization coverage. They however attributed their finding to a lack of timeliness of the birth dose and incompletion of the immunization schedule. The higher prevalence rates of HBV infection among SCA children observed by Jibrin^20^ and Abiodun^28^ et al in Sokoto and Benin respectively contrasted with the index study. This may be explained by the fact that Jibrin et al^20^ excluded children vaccinated against Hepatitis B, whereas the study by Abiodun et al^28^ was conducted before the introduction of hepatitis B vaccination into the National Programme on Immunization in 1997. There was no HBsAg in children below 5 years of age among cases and controls, while the peak prevalence was found in cases aged 13 – 17 years, increased with age, comprising 2% of the 2.5% prevalence found. This increase with age appears to favour horizontal transmission of HBV infection^8^,^13^. This may be attributed to sociocultural practices associated with the adolescents in the study locale as 80% of children with HBV infection engaged in multiple ear piercings. This finding is in agreement with some authors who reported that horizontal transmission of HBV infection is more common than vertical transmission^20^,^29^.

Female adolescents living in rural areas had a higher prevalence rate of HBV infection compared to their male counterparts in the index study, thereby corroborating the report by George and Yaguo^13^. It is possible that these females engaged in invasive procedures like body piercings for various purposes of beautifying themselves commonly carried by native doctors with unsterilized equipment in some communities in the state. This finding is in agreement with that reported by Akpan et al^10^. The prevalence of HCV infection observed in controls was similar to that observed in Enugu state^5^, a state in the same geopolitical zone as the index study. This study observed that a tenth of the SCA children had HCV. This high prevalence may be attributed to the non-availability of vaccines for the virus compared to HBV. Like the HBV infection, the frequency of HCV infection in SCA was observed to increase with age with a median age of 14 years and more in females compared to their male counterparts. Part of the psychosocial developments in mid-adolescence (14-17 years) is the creation of their body image^30^. This may explain the higher percentage of girls with tattoos and ear piercings at this age, and probably the procedures were carried out with contaminated equipment. The high prevalence rate of HCV infection in the index study corroborates the high prevalence rate reported by Jibrin et al in Northern Nigeria^30^ and Mutimer et al^6^ at Benin in Nigeria. This is also similar to the reports by Muhammad et al^29^ and Mehri et al^25^ both studies were done in Saudi Arabia, but in different regions with a prevalence of 12.2% and 12.5% respectively. It however contrasts with the reports by Ejiofor et al^5^ in Enugu who reported a prevalence of 6.6% among children with SCA, Lesi and Kehinde^31^ in Lagos who reported 5%, Aliyu et al^24^ in Zaria which reported a prevalence of 4.4% and Namasopo et al^32^ in Uganda which documented a lower prevalence of 2.5%. Being a female, in the adolescent age group and engaging in multiple ear piercings predicted the prevalence of HCV infection in SCA subjects in the index study. Ear piercings in females are increasingly becoming a fashion trend among teenagers in study locale. This agrees with the findings of some authors^30^ who noted that tattooing and body piercings predicted viral Hepatitis C infection.

## Limitation of study

Inability of the research to determine the maternal HBV and HCV serostatus of the study participants which could be a risk factor for transmission.

## Conclusion

This study showed a high prevalence of HCV infection in children with SCA which was predicted by female gender, age, and multiple ear piercings. The study also highlighted a low prevalence of HBV infection among SCA children and controls, which may be related to increased uptake of routine immunization. Hence, sustained health education, promotion of behavioural change among children with SCA and the general public on risky beautification practices that increase blood-borne infections, and continued sensitization on the benefits of HBV vaccination are recommended.
